# Ultrasound evaluation of penile fractures

**DOI:** 10.2349/biij.7.4.e27

**Published:** 2011-10-01

**Authors:** SG Kachewar, DS Kulkarni

**Affiliations:** Radio-diagnosis Department, Rural Medical College, Maharashtra, India

**Keywords:** Penile fracture, ultrasound, penile trauma

## Abstract

This short case report discusses the various aspects of penile fracture, which is a rare entity. Nevertheless, the incidence of penile fractures is on the rise due to the increased use of performance-enhancing drugs. An individual with a penile fracture should seek immediate medical referral. Prompt diagnosis and management is necessary to prevent undesirable after-effects as discussed. Emphasis is made on how imaging with ultrasound enables a quick and complete assessment of this mishap.

## INTRODUCTION

Penile fracture is defined as the rupture of the tunica albuginea and one or both of the corpora cavernosa. This condition remains under-reported due to ethical and psychological reasons. Diagnosis of penile fracture is mainly based on clinical features. Ultrasound or any imaging is only used when the presentation is atypical. Imaging confirms and evaluates the extent of injury to the tunica.

## CASE REPORT

In a span of one year, the authors saw three cases of penile fractures [Table 1]. Each of the three cases, involving patients aged 47, 32 and 40 years, presented to the Emergency Room with sudden painful swelling and deformity of the penis following sexual intercourse.

**Table 1:** Summary of cases with fractured penis at this institute.

**Parameters**	**Case I**	**Case II** **(Figure 1–4)**	**Case III**
Age in years	47	32	40
Activity-Sexual Intercourse	Yes	Yes	Yes
Use of performance enhancers	Yes	No	No
Pop sound / sensation	Yes	No	No
**Ultrasound Findings**			
1. Tunica albuginea tear	Yes	Yes	Yes
2. Location in shaft	Proximal	Mid-shaft	Proximal
3. Tear orientation	Transverse	Longitudinal	Transverse
4. Size of tear	3 × 5 mm	6 × 2 mm	2 × 2 mm
5. Cavernosal haematoma	Present	Present	Present
6. Urethral Injury	None	None	None
Immediate Surgical Repair Done	Yes	Delayed	Yes
Satisfactory Follow Up at 3-6 month Interval	Yes	No	No
Painful erection	No	Yes	No

The attending casualty medical officer correctly suspected fracture of the penis in all three cases and referred them for emergency ultrasound to confirm the diagnosis. The ultrasound findings are also summarised in Table 1.

Two of these three patients were immediately operated upon successfully and were subsequently followed up at 3- and 6-month intervals, as described in Table 1. One patient deferred surgery due to his initial unwillingness, but later had successful surgery.

## DISCUSSION

Inside a normal penile shaft, erectile tissues are arranged in a columnar fashion. Dorsolaterally, there are two corpora cavernosa, and ventromedially, there is the corpus spongiosum, each enclosed in tunica albuginea. Ventral extension of Buck’s fascia [1, 2] encloses the single corpus spongiosum whereas the dorsal one encloses the two corpora cavernosa.

Rapid rise of pressure inside the corpus cavernosum as a result of an extrinsic force breaks the already thinned (due to erection) tunica albuginea of the erect penis. Activities resulting in an erect state of penis like masturbation, self-manipulation, sexual intercourse, and rolling over in bed, can cause direct penile trauma and result in fractures. Vaginal intercourse remains the most common cause [3]. Increased use of pharmaceutical agents which enhance the duration of erection [4], pre-existing urethral or periurethral infections [5] and injuries to the penis increase the chance of penile fractures.

**Figure 1:**
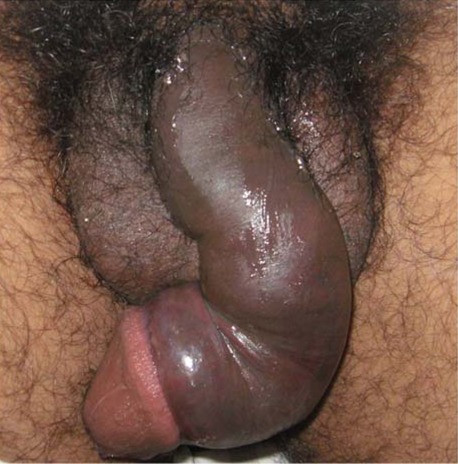
Photograph of a patient with fractured penis on admission.

Afflicted patients usually complain [1–3, 5] hearing of, or feeling, a sudden crack or snap in the penis, together with loss of erection and swollen, deviated, painful penis. Usually only one side of the distal two-thirds of the penis is fractured and less than one-half of the cavernosal circumference is affected. Associated haematomas may be purely intracavernosal or extend to the perineum scrotum, and in some cases, even the thighs. Associated urethral injury presents as haematuria or dysuria.

**Figure 2:**
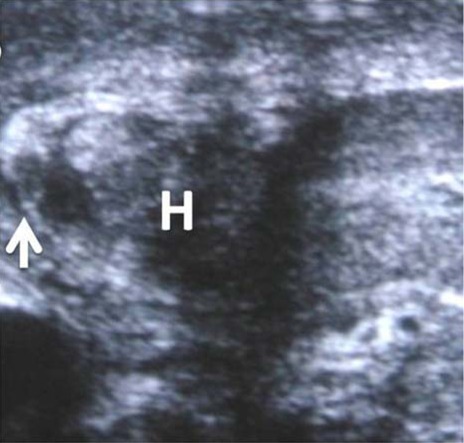
Transverse ultrasound image shows intracavernosal haematoma (H) and the site of ruptured tunica (marked by an arrow).

**Figure 3:**
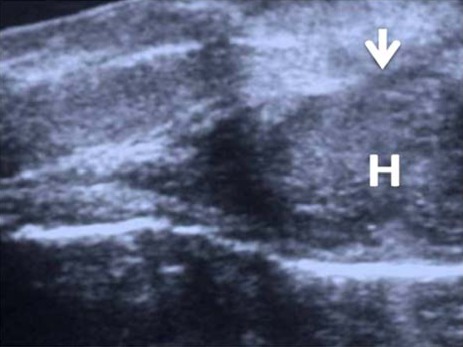
Longitudinal ultrasound image shows intracavernosal haematoma (H) and the site of ruptured tunica (marked by an arrow).

**Figure 4:**
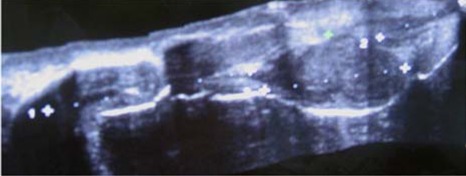
Ultrasound image obtained using crescendo multi-dimensional image processor shows the entire penis from base to tip and site of injury.

**Figure 5:**
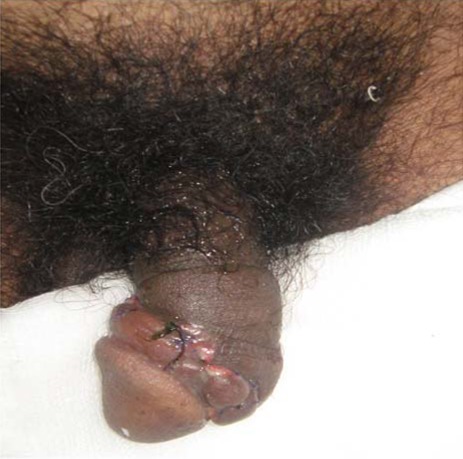
Photograph in post-operative follow-up phase.

Adequate clinical examination may not always be possible or may be inconclusive, hence comes the role of imaging [6]. An array of imaging modalities like cavernosography, ultrasonography (USG) and magnetic resonance imaging (MRI) can be used to identify penile fractures. Though soft tissue details in multiple planes are shown best by MRI, from a practical aspect, USG scores over MRI in terms of cost, availability and time consumed for the procedure [6]. Although it is believed that MRI is superior, insufficient data in the literature suggests that the more practically-available imaging modality (ultrasound) be employed. Early surgical measures involve repair of the torn tunica after haematoma removal and this has been proven to be better than conservative measures [3, 5]. This reduces the chance of angulation deformity that can occur if fibrous plaque is formed due to delay in surgery.

All three patients in our series were followed up for 3–6 months after trauma. Both patients who were immediately treated surgically showed near normal satisfactory erection. The single patient in whom surgery was delayed, due to his initial unwillingness, complained of painful erection.

All doctors must be familiar with the presentation of ‘fractured’ penis, as prompt diagnosis and early surgical repair are the cornerstones to successful outcomes with minimal complications. Public education to seek immediately medical attention, to prevent occurrence of devastating physical, functional and psychological consequences, is the need of the hour.

## CONCLUSION

Penile fractures can be properly diagnosed and treated, provided that doctors are well aware of this entity and only if the patient reports early. Ultrasound provides satisfactory diagnostic details and serves the purpose of imaging in emergency. The fact that USG is non-invasive, cost-effective, involves no radiation and can be repeated even at the bedside any number of times, speaks volumes about its utility as a primary imaging modality in such patients.

## References

[1] Bannister LH, Dyson M, Williams PL (1995). Reproductive system. Gray's anatomy.

[2] Rosse C, Gaddum-Rosse P, Rosse C, Gaddum-Rosse P (1997). Pelvis and perineum. Hollinhead’s textbook of anatomy.

[3] Ruckle HC, Hadley HR, Lui PD (1992). Fracture of penis: diagnosis and management. Urology.

[4] Blake SM, Bowley DMG, Dickinson A (2002). Fractured penis: Another complication of SILDENAFIL. Grand Rounds.

[5] Brotzman GL (1991). Penile fracture. J Am Board Fam Pract.

[6] Nomura JT, Sierzenski PR (2010). Ultrasound diagnosis of penile fracture. J Emerg Med.

